# Estimation of prevalence of mental health problems in 8-10 years old georgian children by using the strengths and difficulties questionnaire*

**DOI:** 10.1192/j.eurpsy.2021.1690

**Published:** 2021-08-13

**Authors:** G. Chvamania, M. Zirakashvili, T. Mikiashvili, N. Mebonia, M. Gabunia

**Affiliations:** 1 Mental Health, Georgian Academy of Childhood Disability, Tbilisi, Georgia; 2 Psychology, Ilia State University, Tbilisi, Georgia; 3 Medical Sciences, Ilia State University, Tbilisi, Georgia; 4 Psychology, Javakhishvili Tbilisi State University, Tbilisi, Georgia; 5 Department Of Chronic Deaseases, National Center for Disease Control and Public Health, Tbilisi, Georgia; 6 Child And Adolescent Mental Health, Mental Health Center, Tbilisi, Georgia

**Keywords:** SDQ, Prevalence in Georgia, Children Mental Health

## Abstract

**Introduction:**

Mental health problems are frequent among children and seems to predict mental disorders in adulthood.

**Objectives:**

The study aimed whether the gender differences affects the Strengths and Difficulties Questionnaire (SDQ) assessments performed by parents and teachers in Republic of Georgia.

**Methods:**

In 2019 a cross sectional survey in four main cities of Georgia was conducted; Totally 8-10 y old 16654 children from 211 public schools were included. SDQ completed by parents and school teachers was used to determine emotional and behavioral problems among Georgian children.

**Results:**

16654 (74%) parents out of 22553 were agreed to participate in the study. 1565 (9.39%) children were rated screen positive in top five percentile by either parent or teacher or both of them. Cut-off scores for 99-95 percentiles (top 1-5%) was defined. Boys were more likely to be rated screen positive than girls, especially by teachers: parents rated screen positive 7.5% of females, teachers - 7.2%, while males 9.4% and 11.5% respectively. Pairwise correlation coefficients (0.53) revealed moderate correlations according to p-values (< 0.05) between scores and all correlations were statistically significant.

**Conclusions:**

The study defined the cut-off scores of SDQ for 8-10 y old children and a gender differences in prevalence of mental health problems in Georgia. SDQ could be used in primary healthcare and school settings to identify children with special needs. This work was supported by Shota Rustaveli National Science Foundation of Georgia (SRNSFG), grant - FR-18-304.

**Disclosure:**

No significant relationships.

